# Infection prevention and control measures for Ebola disease and their outcomes from the perspective of health care workers: a mixed-methods study

**DOI:** 10.1186/s12889-026-27091-z

**Published:** 2026-04-06

**Authors:** Gladys Honein-AbouHaidar, Joanne Khabsa, Victoria Willet, Devika Dixit, Reem Hoteit, Stacey Mearns, April Baller, Elie Akl

**Affiliations:** 1 Hariri School of Nursing, American University of Beirut, Beirut, Lebanon; 2 Global Health Institute, American University of Beirut, Beirut, Lebanon; 3 Clinical Research Institute, American University of Beirut, Beirut, Lebanon; 4 Health Emergencies Programme, World Health Organization, Geneva, Switzerland; 5 Cumming School of Medicine, Department of Pediatrics, Division of Infectious Diseases, University of Calgary, Calgary, AB Canada; 6 UK Public Health Rapid Support Team, London, UK; 7 Department of Internal Medicine, American University of Beirut, P.O. Box: 11-0236, Riad-El-Solh, Beirut, 1107 2020 Lebanon; 8 Department of Health Research Methods, Evidence, and Impact, McMaster University, Hamilton, ON Canada

**Keywords:** Ebola disease, *Orthoebolavirus*, Interest-holders, Health care workers, Perspectives, Preferences, Values, Cost, Equity, Acceptability, Feasibility, Mixed methods, Practice guidelines

## Abstract

**Background:**

This study assessed health care workers (HCWs) perspectives related to eight infection prevention and control (IPC) measures (three personal protective equipment (PPE) and five decontamination and disinfection) for Ebola disease discussed during WHO guideline development.

**Methods:**

We conducted a mixed-methods study guided by the Evidence to Decision framework, targeting HCWs involved in managing patients. We assessed: (1) the value of preventing transmission of *Orthoebolavirus*, adverse effects of PPE and chlorine exposure; (2) perspectives on resource, health equity, feasibility, and acceptability of IPC measures.

**Results:**

Analysis included 73 survey respondents and 16 interviews. Preventing risk of transmission was critical (90%), due to *high fatality*. Side effects of chlorine (49%) and PPE (38%) were critical *due to irritation and heat stress*,* but benefits outweighed harms*. Covering head, neck, and mucous membranes was more acceptable (72%) than covering mucous membranes only as it *enhances safety* but is *more costly* (49%). Use of disposable PPE versus reusable raised equity concerns (62%) due to *supply sustainability*. Goggles under vs. over the hood was less feasible (55%) as they *restrict visibility for those wearing glasses*. Spraying was less acceptable (67%) than wiping as the latter is *more effective*.

Spraying HCWs during doffing was less acceptable (57%) due to *risk of transmission.* Out of four options, removing outer and inner glove was less acceptable (70%) to avoid *exposing* the *skin.* Hand hygiene trade-offs between alcohol-based hand rub (ABHR), chlorine, and soap & water varied, 45% found ABHR more acceptable, *less pungent and kills the virus*,* but* is resource-intensive (58%). Incineration was more acceptable (59%) and feasible (58%) than disinfection, but *incinerator is costly.*

**Conclusion:**

This study highlighted variability in acceptability, feasibility, and equity concerns for IPC measures. Discussion of those findings during guideline development was crucial to increase applicability and acceptability by end users.

**Supplementary Information:**

The online version contains supplementary material available at 10.1186/s12889-026-27091-z.

## Background

Ebola disease (EBOD) is a severe illness with an average case fatality rate of approximately 50% [1], especially in the absence of vaccination or treatment. EBOD is caused by *Orthoebolavirus.* Historically, *Orthoebolavirus* is introduced into humans through animal-to-human (zoonotic) transmission with subsequent human -human transmission through contact with blood and body fluids of infected individuals [2, 3]. This requires the use of contact and droplet (due to the risk of splashing or spraying of body fluids) precautions including the associated PPE.

The 2014–2016 West African EBOD outbreak marked the largest and most protracted outbreak of *Orthoebolavirus* [1]. Since then, several other large outbreaks have occurred.

Health care workers (HCWs) are particularly at risk for the disease and are a potential risk for its transmission. Infection prevention and control (IPC) is a cornerstone in *Orthoebolavirus* outbreak response. HCWs infection due to poor adherence to IPC measures has been reported [4]. During the 2014–2016 West African outbreak, national HCWs were disparately infected, likely associated with insufficient personal protective equipment (PPE) or inadequate decontamination and disinfection measures [1]. Savini et al. documented several occupational exposures among HCWs in an Ebola treatment center in Conakry, Guinea, most commonly from goggles or respirator masks slipping during patient care [5]. Although 80% of exposures were classified as low risk and no secondary infections occurred, the study highlighted persistent vulnerabilities in PPE performance and biosafety compliance despite comprehensive training. Additionally, Selvaraj et al. in a systematic review of Ebola and Marburg virus outbreaks, identified insufficient or incorrect PPE use, exposure to unrecognized infected patients, inadequate hand hygiene, and deficiencies in infection control infrastructure and training as the most frequent contributors to HCW infection [6].

During and following the 2014–2016 West African outbreak, the World Health Organization (WHO) issued several interim/rapid advice IPC guidance documents for HCWs in contact with EBOD patients [7–9]. Since then, several factors prompted the WHO to re-evaluate and update previous guidance [3]. These included a growing evidence base and HCWs experience implementing IPC measures, vaccines development [10], and new practices emerging from the field in the context of further outbreaks (e.g., IPC ring approach) [11]. In light of this, in 2021 the WHO IPC Ebola guideline development group (GDG) was established. The GDG defined questions for the guideline, cumulating in prioritization of eight IPC measures for mitigating *Orthoebolvirus* transmission [12]. First, systematic reviews were conducted to identify evidence on the health effects of the eight IPC measures of interest [13]. Second, an assessment of HCWs’ perspectives on those measures was conducted to inform the GDG deliberations. The latter was particularly pertinent when there was limited or no evidence base resulting in low or very low certainty of evidence [3] and to optimize uptake of recommendations [14]. Following this, the GDG met multiple times between 2021 and 2022 to review evidence, HCW survey results and formulate IPC recommendations, following the Grading of Recommendations Assessment, Development and Evaluation (GRADE) methodology [15].

The objective of this study was to assess HCWs’ perspectives on the eight proposed IPC measures for EBOD. The findings were presented at the GDG meeting to assist in the deliberations of the WHO guideline development.

## Methods

### Study design

A parallel mixed-methods study design was adopted and included an online survey and in-depth interviews, guided by the Evidence to Decision (EtD) framework [16, 17]. The complementary nature of using both methods provides a more comprehensive and nuanced understanding of the perspectives of interest holders on IPC measures. The triangulation of results can either converge, diverge, or expand this understanding. Data was collected between September and December 2022. The guideline on Good Reporting of A Mixed-Methods Study (GRAMMS) was followed [18] (Appendix 1: Good Reporting of A Mixed-Methods Study (GRAMMS)).

### Theoretical framework

The EtD framework includes important criteria that should be considered by a GDG when developing recommendations, including valuation of outcomes and contextual factors (i.e., resources needed, impact on equity, feasibility, and acceptability of the interventions) [16, 17] (Appendix 2: Description of EtD framework).

### IPC measures

The eight IPC measures of interest to the GDG were grouped into two categories: PPE; and decontamination and disinfection. (Appendix 3: Comparisons of interest for the WHO guideline).

### Study population and sampling

The targeted population consisted of HCWs involved in supporting EBOD outbreak responses in countries such as the Democratic Republic of Congo, Uganda and West African countries.

Convenience sampling methodology was used to recruit participants for the online survey. Members of the GDG, implementing partners, WHO secretariat, United Nations (UN) agencies staff, non-governmental organizations such as Médecins Sans Frontiers (MSF) were asked to disseminate the survey within their network and with individuals who met the following inclusion criteria: decision-makers at the Ministry of Health of respective countries or HCWs directly or indirectly involved in the management of patients with EBOD in routine heath facilities or Ebola treatment centers (ETCs).

The online survey included a consent form in which respondents were asked whether they would be willing to participate in a follow-up in-depth interview and to provide their contact information. Participants who expressed interest were subsequently invited to take part in the qualitative interviews. Of the 73 individuals who completed the survey, sixteen consented and were interviewed for the qualitative component.

### Patient and public Involvement

Patients and the public were not involved in the conduct, design, or results of this study.

### Data collection

Like previous studies informing WHO guidelines [19–25], the survey was developed in English and French. The questions reflected the proposed recommendation questions that were intended to be discussed at the guideline development group meeting. To ensure that healthcare workers’ perspectives could directly inform WHO guideline deliberations, we used the constructs of the Evidence to Decision (EtD) framework. The survey was piloted with two WHO HCWs who met the eligibility criteria to examine the clarity of the questions and make the final amendments accordingly (Appendix 4: Online survey instrument). Given that the primary objective was to characterize the distribution of perspectives across contextual domains, we did not pursue the psychometric properties of the survey. Microsoft Dynamics 365 Customer Voice [26] was used to administer the online survey including consent form, between September 22 and October 17 2022.

An interview guide was developed covering the same constructs as the survey but seeking deeper perspectives (Appendix 5: Interview guide). Interviews were conducted via Zoom audio-conferencing between October and December 2022 in either English or French, according to participant preference, by a bilingual researcher fluent in both languages. All interviews were audio-recorded and securely stored on the main investigator’s computer. All recordings and transcripts were pseudo-anonymized using a unique identifier per interviewee, e.g. P01. Qualitative data were analyzed in the language in which they were collected to preserve meaning, and no prior translation was required.

### Data analysis

#### Survey analysis

Using Microsoft Excel, survey responses were analyzed using frequencies and percentages. The quantitative analysis was limited to descriptive statistics, as the primary objective was to characterize healthcare workers’ perspectives to inform WHO guideline deliberations using the Evidence to Decision (EtD) framework rather than to test hypotheses or examine subgroup differences. Given the convenience sampling approach and the absence of a defined sampling frame, the study was not powered for inferential analyses, and no a priori power calculation was conducted.

Responses to the valuation of outcomes were classified into three categories: critical (ratings of 7–9), important (ratings of 4–6), and less important (ratings of 1–3), following the GRADE guidance on the subject [27]. For contextual factors, the responses for resources, acceptability, and feasibility were classified into three: ‘more, less, negligible, varies, don’t know’. Equity was dichotomous: ‘yes, no’. Responses to open-ended questions in the survey were added to the interview data and analyzed together.

#### Interview analysis

For qualitative data analysis, the framework method for analysis and thematic analytical approach based on 7 stages was used [28] ensuring credibility, reflexivity, and confirmability throughout the process. For credibility and confirmability, transcribed audio-recorded interviews as the main data repository and a semi-structured interview approach were used. For reflexivity, the interviewer had no prior relationship with participants, and two individuals were involved in the analysis to avoid interpretation bias (Appendix 6: Framework thematic analysis).

### Mixed methods integration

This study used a convergent mixed methods research design [29]. Merged findings from quantitative and qualitative analysis were compared and further analyzed for concordance, discordance or expansion. A contiguous approach to integration was followed, where results from each item were presented separately within the same report before being jointly interpreted [29].

## Results

### Participants’ characteristics

Seventy-three individuals participated in the survey. Fifty-six (56%) of responders were males and 44% female, comprising physicians (48%) and nurses (26%), and were employed in health-care facilities (40%), governmental organizations (30%), or non-governmental organizations (30%). The majority of participants provided direct care to EBOD patients (59%), mainly in Ebola treatment centres (ETC) (56%). Appendix 7 presents the survey participants’ characteristics.

A total of 16 individuals were interviewed, 10 provided direct clinical care and 6 were IPC trainers. Roles included: 2 IPC officers, 4 nurses, 3 doctors, 2 medical laboratory, and 5 public health specialists.

After 16 interviews, we reached code and meaning saturation. Code saturation was achieved once all major perspectives on each measure had been identified and no new issues emerged, while meaning saturation was achieved when these perspectives were understood in-depth [30]. Two researchers conducted the analysis, allowing us to confidently confirm that both types of saturation had been attained.

### Valuation of outcomes results

1. Preventing *Orthoebolavirus* transmission is critical
  Most participants rated HCWs *Orthoebolavirus* transmission prevention as critical (90%) due to the high fatality rate, high risk for occupational and health care -associated infection, and significant public health hazard if the virus is transmitted to community members. *Orthoebolavirus* transmission among HCWs has dire consequences on the health care system given the scarcity of HCWs providing EBOD services, losing workforce members puts the health care system at risk: *“because if health care workers are not going to work. that’s another big problem”* (P13). Secondly, if the measures fail to stop transmission, *“the confidence of the people [in the health care system] decreases/wanes?”* (P12).
2. PPE adverse effects are critical and important
  Those who rated PPE adverse effects as critical (38%) and important (28%) indicated that as EBOD is predominately prevalent in hot climate countries, *“the heat and dehydration can make the person wearing the PPE faint”* (P02) and *“the sweat inside the goggles can make it difficult to see and increases the risk of needle stick or scalpel injury”* (P12).
  Those who rated PPE adverse effects as less important (34%) indicated that PPE is not the problem, but rather *“abiding to the protocol in terms of how much time is spent in the PPE*,* and using quality materials*,* which were the major problem”* (P14).
3. Chlorine adverse events are serious matters
  Chlorine at specific concentrations is used to disinfect health care facility surfaces. Most participants rated the adverse effects of chlorine as critical (49%) or important (33%).
  The corrosive nature of chlorine “*is tough on people”* (P02), and its repetitive use can lead to *“airways disease or asthma and induce dermatitis”* (P10). Further, *“the major concern is that often the chlorine concentration used in ETC is not 0.05% but rather 0.5% and sometimes it is powdered chlorine*,* a highly corrosive substance”* (P13).
4. Comparison between *Orthoebolavirus* transmission, PPE, and chlorine adverse events
  More than half of participants were more concerned about *Orthoebolavirus* transmission than the adverse effects of PPE (53%), and chlorine exposure (58%). Participants indicated that EBOD is often fatal, while adverse events of PPE and chlorine are less serious, transient, and not transmissible.

*“The horribleness of having Ebola and what people can go through… also*,* transmission is dangerous*,* while I can’t transfer chlorine exposure”* (P03).

Also, the benefits of PPE and of chlorine use in terms of prevention of transmission were perceived to outweigh their harms.

*“I will bear the side effects of the chlorine exposure to protect myself because there is no actual medication for Ebola. Once you get the virus*,* it’s all about your body to fight. I’d rather have this short-lived effect then ending my life”* (P08).

The remaining participants were equally concerned about EBOV transmission and the adverse events of PPE (47%) and chlorine use (42%), specifically that inappropriate PPE use can lead to disease transmission.

*“It is major discomfort… It is horrible… It is occluding my sight. Even dealing with little things… You want to touch your face… I find it extremely uncomfortable to deal with those adverse events… At times*,* I am at more risk because I am messing up with my PPE. I have done things that shouldn’t have been done”* *(P03).*

More quotes are included in Appendix 8.

### Contextual factors result

**Fig. 1:**
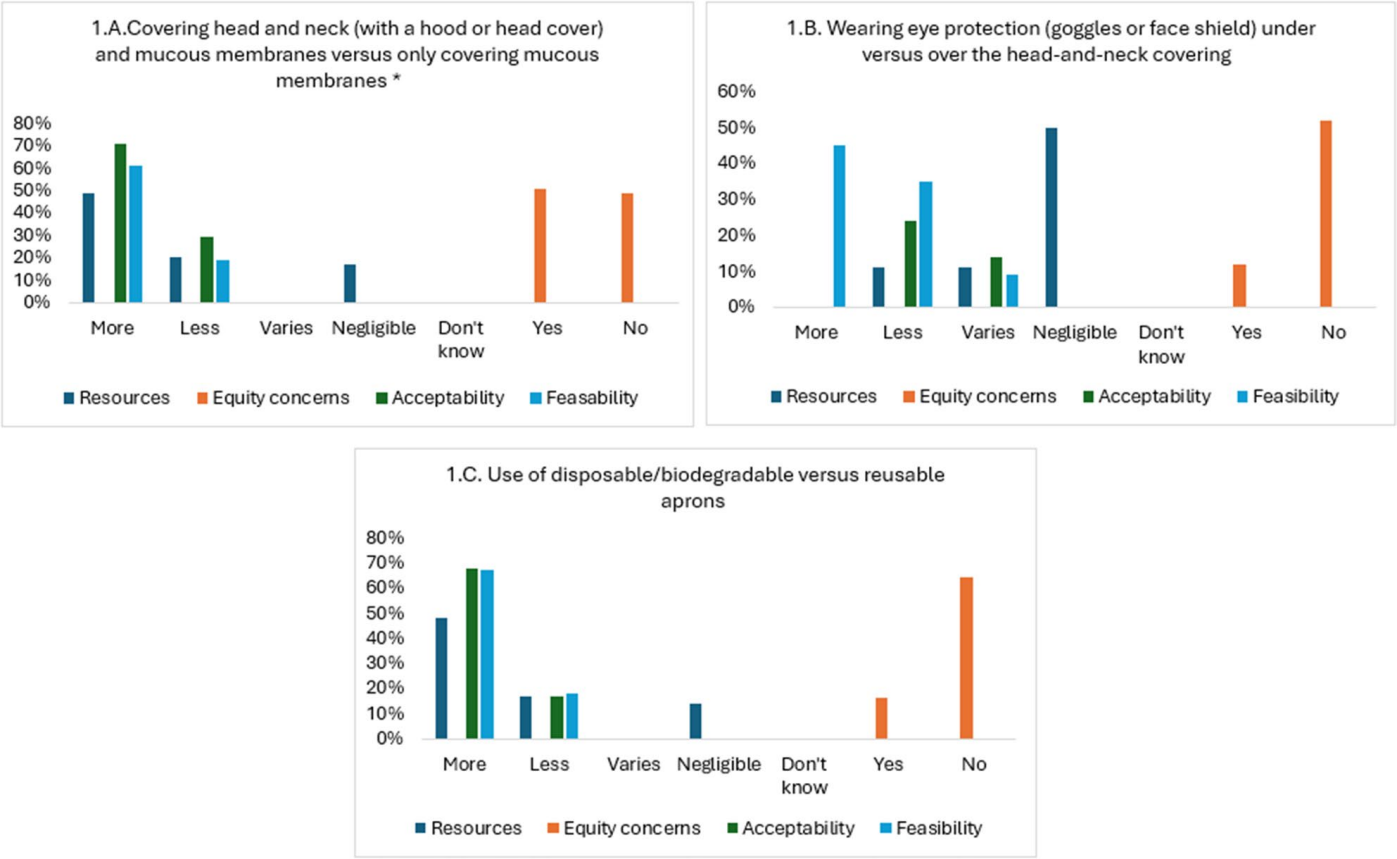
**A**-**C** Summary of survey results for contextual factors related to PPE use

**Fig. 2:**
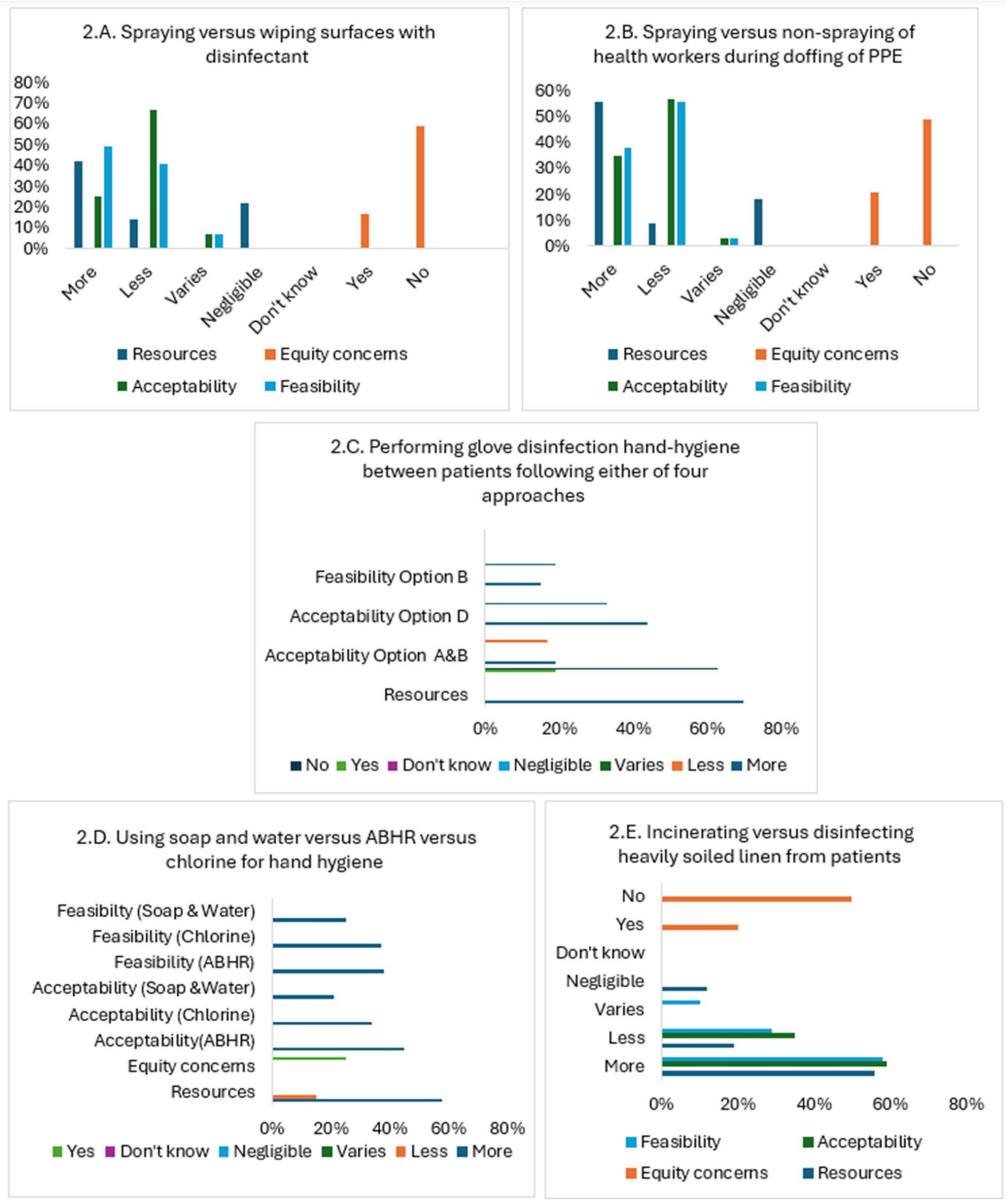
**A**-**E** Summary of survey results for contextual factors related to decontamination and disinfection practices

IPC measures were grouped into two categories: (i) PPE use and (ii) decontamination and disinfection. HCW ‘s perspectives on contextual factors including resources, equity, acceptability, and feasibility for each of the two categories are reported in Figs. 1A-C and 2A-E respectively. Please refer to Appendix 3 for the comparative IPC measures reported in those figures. Selected participant quotes for each finding are provided in Appendices 9 and 10.

i. PPE use
  Participants felt that the covering head, neck and mucous membranes vs. just covering mucous membranes when caring for patients with EBOD was generally of no concern in terms of equity (52%), more acceptable (72%), and more feasible (61%) but may increase costs (49%). Participants indicated they felt safer with this coverage and found donning the PPE easy *“The head-and-neck covering ensures complete protection and enhances the feeling of safety”* (P01).
  For eye protection (goggles, face shield) under versus over the hood, HCWs reported negligible difference in resources (50%), since the same PPE was being used in both scenarios, no equity concerns (52%) but variations in terms of acceptability (49% more acceptable) and feasibility (45% more feasible) *“Wearing goggles under the head covering can be quite uncomfortable for those who need glasses because the goggles can press against the glasses and restrict visibility”* *(P10)*. Wearing eye protection under the hood was perceived as providing a higher sense of safety.
  The use of disposable/biodegradable versus reusable aprons was reported as more costly (48%), no concerns with impact to equity (64%), and generally more acceptable (68%) and feasible (67%) to implement. *“The reusable ones need cost for cleaning*,* and the disposable ones need consistent restocking”* *(P02)*. HCWs described disposable aprons as more comfortable, easier to manage, perceived higher level of safety and reduced exposure to chlorine.
ii. Decontamination and disinfection practices
  HCWs identified spraying of surfaces compared to wiping as requiring more resources (42%), as feasible (49%), but less acceptable (67%) as wiping was perceived to be more effective in hard-to-reach areas, stays longer on the surface, and in anyway *“If something is contaminated with blood*,* you don’t spray*,* you wipe”* *(P01)*. Two third of participants identified no equity concerns (59%).
  Spraying HCWs during doffing of PPE vs. non-spraying required more resources (56%), generally less acceptable (57%), and less feasible (56%). Although 49% identified no equity concerns, 21% did raise concerns for individuals with existing health problems (e.g. respiratory problems, allergies) *“It makes people sick*,* so you are losing people at work”* *(P03)*.
  Most survey participants (70%) indicated that removing the outer and inner glove, washing/disinfecting the hands, and putting on new gloves was associated with the highest resources (Appendix 3: Comparisons of interest for the WHO guideline). *“The inner glove is like a second skin. When you remove the inner glove*,* your skin is exposed”* *(P01)*. option (d) was the most acceptable and options (a) and (d) were equally feasible (33%), equity concerns were cited by 19%.
  Trade-offs between ABHR, chlorine, and soap & water for hand hygiene varied. ABHR was the most acceptable (45%) as “*the virus is an envelope virus so the alcohol will kill the virus*_*(P06)*_ followed by chlorine (34%), then soap and water (21%). But, ABHR is resource-intensive (58%) and is often reserved for well-resourced settings. Chlorine was deemed highly feasible by 37% of participants due its ease of use, but has some challenges such as potential skin irritation.
  For heavily soiled linen, incineration was more acceptable (59%) and feasible (58%) than disinfection due to its perceived effectiveness and simpler implementation, but it is costly and resource-intensive (56%), as buying “*an incinerator is a huge money”* *(P04)* and need replenishing of linen. Disinfection is less resource-intensive and more economical but raises concerns about staff safety *“There are high risks of individuals who are handling those linens”* *(P07)*.

## Discussion

The findings from our initial work indicate that prevention of EBOD transmission among HCWs through IPC measures was perceived as a critical outcome, given the high fatality rate and potential consequences for the health care system. Reducing the adverse events related to chlorine and PPE use were important outcomes but were considered less serious than the risk of EBOV transmission. This study also reported on several contextual factors that influence the uptake of EBOD IPC measures. It was of utmost importance that these factors were considered during guideline development. If guidelines contain recommendations that are challenging to adhere to due to contextual reasons, they risk lack of or incomplete implementation and thereby rendered ineffective.

### Contextual factors affecting guideline implementation

The contextual factors mainly determined by the socioeconomic factors in the country impacted availability of resources, specifically PPE, disinfectant, waste management, and water supply. Due to the limited financial capacity, access to supplies, such as PPE, both in quantity and quality, was consistently reported as a major resource challenge [5]. Participants indicated that the supplies were limited, and the supply chain was at times not sustainable.

Further, the differing quality of PPE, in spite of existing WHO recommendations outlining PPE product technical requirements for viral hemorrhagic fevers contributed to poor acceptability, feasibility, and equity [31]. Women wearing a hijab and those with voluminous hair may find the PPE to be of poor fit hence influencing feasibility and acceptability. Similar findings were echoed in other studies where poor compliance to IPC measures was associated with availability, quality, and fit of PPE [32–35]. Some participants described the PPE as inducing fear in patients and impacting communication ability. Survey participants also described concerns with poor fit of some PPE (hoods, aprons) that may lead to contamination risks. Fogging associated with eye protection leading to poor visibility and challenges for individuals wearing glasses were also described. Lastly, concerns with appropriate decontamination of reusable PPE were also noted [34, 35].

Health facility infrastructure in resource limited settings was identified as a major factor impacting feasibility and equity. Insufficient water supply led to inability to consistently comply with hand hygiene requirements [36]. Waste management, (i.e., storage, transportation and final disposal) requires facilities to properly dispose PPE and other biomedical waste (e.g. incinerators), and those are scarce in resource limited health facilities. Further, where they exist, they are costly to run, requiring consistent electricity and technical expertise [34, 35]. These concerns have been raised in other publications including the need to reduce waste [37, 38].

While spraying of surfaces for disinfection and of HCWs during PPE doffing protocols in EBOD outbreaks has been a common practice, survey participants identified numerous concerns impacting acceptability, feasibility and equity of these practices. While some indicated a perception of increased safety with spraying since it increases coverage of surfaces and penetrates of cracks, others suggested that wiping is often a required first step and may be more effective in hard to reach area. As for spraying HCWs during doffing, most participants did not perceive its added value. They were concerned with the potential risk of contamination if mucous membranes were not protected. It required specialized devices, guidance and dissemination on appropriate preparation and chlorine use.

Socio-ecological factors also played a role including weather and religion. Most EBOD incidence occurs in hot, tropical climates. The heat in the ETCs and HCW sweating while using PPE were major challenges for accepting IPC measures and added safety concerns. A few participants refused the use of alcohol-based handrub (ABHR) due to religious beliefs.

Additional factors that impacted glove decontamination and hand hygiene acceptability, feasibility, equity and resources included frequent changing of gloves requiring high consumption of the product, leading to supply challenges. Glove allergies, and impracticality of double gloving making simple tasks more difficult were described. Challenges with putting new gloves over wet inner gloves were described as well as fears of possible virus transmission if changing gloves within the ETC or weakening of gloves due to decontamination (disinfectant use) protocols.

### Strengths and limitations

This study provided original findings and reports on challenges that HCWs directly or indirectly involved in EBOD patient care encountered when implementing IPC measures. The study’s exploratory qualitative arm identified findings that would not have been possible to extrapolate from the survey alone. More specifically, in situations where evidence is limited or absent, considering interest-holders’ perspectives on the contextual factors assisted the guideline development group in formulating their judgements and recommendations [3, 39, 40]. GDG members carefully reviewed all data, finding the concrete numbers and quotes exceptionally useful for informing and making critical judgements. This input was highly valued by the GDG, particularly in informing recommendations where evidence was of limited availability or quality, sparking important discussions, and allowing them to make more informed and robust recommendations.

The study limitations involved the sampling and data collection processes. We did not have access to reliable data on the total number or geographic distribution of HCWs involved in EBOD outbreak response across the targeted countries. Consequently, it was not possible to establish a sampling frame, determine country-level response rates, or assess representativeness. Given this constraint, recruitment relied on convenience sampling through professional networks and partner organizations operating in Ebola-affected settings. The use of a convenience sample limits generalizability of the findings and may have introduced self-selection bias as those who opted to participate may differ from the rest of the targeted population, and reduced the ability to control for the sample characteristics. The recall bias associated with collecting data based on their experience dating back a few years and the social desirability bias may also have impacted the credibility of findings. Finally, the length of the online survey, with 36 structured questions accompanied with open-ended comments, may have led to respondent fatigue.

While the mixed-methods design was employed to achieve a complimentary approach to have an in-depth understanding of the HCWs perspectives on the IPC measures, the specific methodological limitation that must be acknowledged is the interpretation bias. Integrating quantitative and qualitative findings relied heavily on the researchers’ interpretive decisions. Choosing a specific statement to explain a numerical finding was a subjective decision, hence arbitrarily rather than absolute certainty.

### Implications for practice, research, education

In terms of practice, this study raised two important concerns for potential HCWs risks that may occur due to common IPC practices: chlorine exposure and prolonged PPE use adverse events. First, spraying of chlorine and the adverse events of chlorine exposure during an EBOD outbreak reported by survey participants and other literature [41] are important as they cause eye, respiratory, and skin conditions. Second, some risk factors contributing to adverse effects to HCWs from prolonged PPE use reported have been identified in other studies [42, 43] and include pain in the back of the ear, pressure related injury, fogging and extreme sweating. These findings highlight areas that may benefit from continuous quality improvement initiatives and closer monitoring in outbreak settings, particularly regarding chlorine preparation and PPE workflow practices [44], especially in challenging climates. Accordingly, our results underscore the importance of ongoing evaluation of implementation challenges to support safer IPC practice. Additionally, lack of training on risk assessment and PPE rationalization, limited handwashing with soap and water or reluctance to use ABHR suggest opportunities to strengthen training approaches, including emphasis on the “do nots” alongside proper IPC protocols [45].

As for research, this study highlighted a significant gap in the literature regarding the adverse effects of PPE, particularly in tropical climates. The recent COVID-19 pandemic triggered a wave of studies focusing on the adverse events of PPE [35, 46, 47]. Similar to this study, the prolonged use of PPE was an important factor for adverse events [48], however none of the studies focused on climatic factors that can exacerbate these adverse events. This underscores the need for more research on PPE design, particularly for tropical climates, appropriate adherence to protocols, i.e. time spent in PPE, fit and size, and the overuse of PPE based on fear and misinterpretation of evidence. PPE and use of chlorine for decontamination has been set as a research priority by WHO [49]. Furthermore, future studies need to build on the findings of this study, for example, using implementation science, research can monitor the implementing of each measure and identify the fidelity challenges and their outcomes. Importantly, given the complexity and high-risk nature of Ebola outbreak settings, there is a need for more in-depth qualitative and theory-informed research to better understand HCWs’ lived experiences, contextual constraints, and adaptive practices within these difficult working environments.

In terms of methods, our survey improved upon previous efforts on the discrimination ability of the survey to differentiate between the different ratings from those used in previous similar surveys [19, 21, 22]. The questions used simple wording, and definitions were provided for each construct. However, to align with the purpose of the study, in future studies, questions should clearly ask for participants’ perceptions by prefacing with: ‘Do you believe that …? Please provide the response that reflects your view”. Also, to maintain a consistent polarity from strongly disagree to strongly agree, a neutral question should be included in the middle e.g., Strongly disagree (not more acceptable), Disagree (probably not more acceptable), Neutral (no difference), Agree (probably more acceptable), Strongly Agree (definitely more acceptable), and include Not sure/Don’t know option.

As for education, our findings reveal a need to improve the education and training of HCWs on IPC measures as there is a disconnect between protocols and practice. Studies found that HCWs’ awareness of transmission and IPC measures was suboptimal even in other countries that witnessed EBOD epidemics [50–52], such as Nigeria, where EBOD and its transmission knowledge was high (72.5%), yet the level of good practices was suboptimal. These findings reinforce the importance of targeted, evidence-informed training, including emphasis on inappropriate PPE use and other “do nots.” Continuous education and quality improvement strategies may help response teams better understand and address ongoing challenges related to chlorine exposure and prolonged PPE use in outbreak settings.

## Conclusion

This study was integral to the GDG discussion in the formulation of the WHO Infection prevention and control guideline for Ebola and Marburg disease update given the paucity of other evidence. It highlighted the perceived critical role of preventing EBOV transmission among HCWs. It also showed variability in acceptability and feasibility of IPC measures for EBOD and shed light on equity concerns. By addressing contextual factors influencing implementation, guidelines are more applicable and acceptable to end users.

## Supplementary Information

Supplementary Material 1.

## Data Availability

All relevant data are within the manuscript and its Supporting Information files.
