# Supplementary material for: Infection prevention and control measures for Ebola disease and their outcomes from the perspective of health care workers: a mixed-methods study
Source: BMC Public Health. 2026 Apr 6;26:1581. doi: 10.1186/s12889-026-27091-z (PMC13191939; doi:10.1186/s12889-026-27091-z)
Supplement: Supplementary file 1 — Supplementary Material 1. [file 12889_2026_27091_MOESM1_ESM.docx]

**Infection Prevention and Control Measures for Ebola Disease and their Outcomes from the Perspective of Health Care Workers: A mixed-methods study**

## **Appendix 1:** Guideline for Good Reporting of A Mixed-Methods Study (GRAMMS)

| GRAMMS Reporting Criteria | Your Response |
| --- | --- |
| 1. Justification for using a mixed methods approach: Explain why both qualitative and quantitative methods were needed. | A justification for using a mixed-methods approach was reported in the “Study Design” section. In brief, the complementary nature of using both methods provides a more comprehensive and nuanced understanding of the perspectives of interest holders on IPC measures |
| 2. Description of the mixed methods design: Describe the type of design (e.g., sequential, concurrent, embedded) and the priority of each method. | The type of design was reported in the “Study Design” section. It stated that a parallel mixed-methods study design was adopted, hence no priority statement is required. |
| 3. Detailed description of each method: Explain the sampling, data collection, and analysis for qualitative and quantitative components. | Under Methods, the sampling, data collection, and data analysis are clearly stated. |
| 4. Description of integration: Indicate where and how integration occurred (e.g., during design, analysis, interpretation). | Integration was described under Methods. The integration occurred during the analysis and interpretation. |
| 5. Limitations of integration: Describe any challenges or limitations related to integrating the methods. | The limitation of integration was reported under the sections of “Strengths and Limitations” |
| 6. Insights gained from integration: Explain the added value or new understanding gained from combining methods. | The added value has been reported under the justification for using mixed-methods approach under the section of “Study Design” |

## **Appendix 2:** Description of EtD framework

The EtD framework consists of the following constructs:

Valuation of outcomes

For the valuation of outcomes, the below three main outcomes were prioritized by the GDG and were considered: transmission of *Orthoebolavirus*; adverse effects of personal protective equipment (PPE) use; and adverse effects of chlorine exposure. For each of the outcomes, we developed health outcome descriptors (18) to explicitly define them and to ensure a common understanding among participants.

Contextual factors of interest

- Resource use: the resources associated with deploying a certain intervention, including: (1) health-care resources (e.g., costs of the intervention, human resources, time, hospital visits and home visits); (2) non-heath care resources (e.g., social welfare services); and/or (3) patient and informal caregiver resources (e.g., time of family).
- Impact on health equity: how the implementation of a guideline recommendation may impact certain patients/people from having equal and fair chance to receive the required intervention. Subgroups for whom equity might be particularly relevant are in relation to their place of residence, race/ethnicity/culture/language, occupation, gender/sex, religion, education, socioeconomic status, and social capital (19).
- Acceptability: the perception of whether an intervention is appropriate, agreeable, tolerable, or satisfactory. Acceptability may be affected by the characteristics of the intervention as well as the person’s culture, preferences, beliefs and experiences related to the intervention.
- Feasibility: extent to which an intervention can be successfully implemented within a given setting. Feasibility may be affected by the barriers and facilitators to implementing the interventions, resources needed (e.g., human resources), sustainability, availability, accessibility, and the potential for integration of an intervention within an existing health program.

## **Appendix 3:** Comparisons of interest for the WHO guideline

**I.PPE use:**

I.A. Covering head and neck (with a hood or head cover) and mucous membranes versus only covering mucous membranes.  (

I.B. Wearing eye protection (goggles or face shield) under versus over the head-and-neck covering

I.C. Use of disposable/biodegradable versus reusable aprons

**II. Decontamination and disinfection:**

II.A. Spraying versus wiping surfaces with disinfectant

II.B. Spraying versus not spraying of health-care workers during doffing of PPE

II.C. Performing glove disinfection in-between patients following either:

1. Washing/disinfecting the outer glove
2. Washing/disinfecting the outer glove, removing it, and putting on a new glove
3. Washing/disinfecting the outer glove, removing it, washing/disinfecting the inner glove, and putting on a new outer glove
4. Removing the outer and inner glove, washing/disinfecting the hands, and putting on new gloves

II.D. For hand-hygiene using which of the following:

- Soap and water
- Alcohol based hand-rub (ABHR)
- Chlorine

II.E. Incinerating versus disinfecting heavily soiled linen from patients

## **Appendix 4:** Online survey instrument

### Part 1: Outcomes valuation

The outcomes of interest for a recommendation question consist of the efficacy and safety outcomes potentially affected by at least one of the alternative interventions being considered. Examples of efficacy outcomes could include mortality, morbidity, transmission of disease, and person-reported outcomes. Examples of safety outcomes could include adverse events, serious adverse events, and any other unintended effects.

The purpose of this survey is to rate the importance of outcomes for making a recommendation related to infection protection and control measure in the context of an Ebola disease outbreak, where:

- Outcomes scored 7-9 will be considered as ‘critical’ for making the recommendation, i.e., the evidence for these outcomes will drive the decision making. Also, the overall certainty of evidence for the recommendation will depend on the certainty of evidence for these outcomes;
- Outcomes scored 4-6 will be considered as ‘important’ for making the recommendation, i.e., the evidence for these outcomes will inform but not drive the decision making;
- Outcomes scored 1-3 will be considered ‘less important’ for making the recommendation, i.e., the evidence for these outcomes may not be considered (e.g., will not be included in the evidence profile).

The outcomes of interest considered in this study include:

- Transmission of Ebola
- Adverse effects from chlorine exposure
- Adverse effects from PPE use:
  - Heat intolerance
  - Dehydration
  - Discomfort
  - Environmental impact of single-use disposable PPE

Before rating the importance of each outcome, please consider the following health outcome descriptors.

*Outcome 1: Transmission of Ebola*

Health workers may become infected with Ebola whilst at work if IPC measures are not followed correctly. The outcome of EVD exposure is dependent on several factors including access to EVD vaccination, and if infected, access to therapeutics which can lower mortality. The mortality from infection without access to therapeutics on average is 50%.

- **Symptoms:**
  - Early symptoms of fever, headache, and weakness are followed by severe gastrointestinal symptoms (severe watery diarrhea, nausea, vomiting, and abdominal pain)
  - Symptoms may progress to multiple organ dysfunction and death
- **Time horizon:**
  - Patients infected with Ebola will develop symptoms within 21 days of exposure (incubation period).
  - Patients can progress from the initial nonspecific symptoms to gastrointestinal symptoms in about 5 days.
  - Death may occur between days 6 and 16 following the onset of symptoms.
- **Testing and treatment:**
  - A polymerase chain reaction (PCR) blood test is used to detect the virus.
  - The patient is isolated and treated in Ebola Treatment Units.
  - Treatment typically includes administration of monoclonal antibodies, and medications to treat symptoms including medication for blood pressure, vomiting and diarrhea, fever and pain.
- **Consequences:** Case fatality rates have varied between 25% to 90% in past outbreaks

*Outcome 2: Adverse effects from chlorine exposure*

Health worker exposure to chlorine through environmental disinfection and decontamination when removing PPE. Exposure more likely to occur with spraying of chlorine compared to other methods such as wiping.

- **Symptoms:**
  - The main symptoms of acute exposure to chlorine are related to breathing (tightness of the chest, respiratory distress), vision (visual disturbance, loss of visual acuity), and skin (burning and dermatitis).
  - Even a single exposure can lead to symptoms.
  - Multiple exposures are associated with an increase in symptoms.

*Outcome 3: Adverse events from PPE use*

Additional layers of PPE worn by health workers may lead to several short-term negative effects:

- **Symptoms:**
  - Discomfort
  - Dehydration
  - Heat intolerance
- **Consequences:**
  - Can negatively impact communication with patients
  - Difficulty performing patient care duties

1. Referring to the health descriptors described above, please rate each of the following outcomes on a scale of 1 to 9, with 1 being the least ‘important’ and 9 being the most ‘critical’ for making the recommendation:

| **Outcome** | **Not important** | | | **Important** | | | **Critical** | | |
| --- | --- | --- | --- | --- | --- | --- | --- | --- | --- |
| 1. EVD transmission | 1 | 2 | 3 | 4 | 5 | 6 | 7 | 8 | 9 |
| Please provide an explanation for your rating |  | | | | | | | | |
| 1. Adverse effects from chlorine exposure | 1 | 2 | 3 | 4 | 5 | 6 | 7 | 8 | 9 |
| Please provide an explanation for your rating |  | | | | | | | | |
| Are you more concerned about adverse effects from chlorine exposure or EVD transmission? | 1. I am more concerned about adverse effects from chlorine exposure than EVD transmission 2. I am more concerned about EVD transmission than adverse effects from chlorine exposure 3. I am equally concerned about EVD transmission and adverse effects from chlorine exposure | | | | | | | | |
| Why? |  | | | | | | | | |
| 1. Adverse effects from PPE use | 1 | 2 | 3 | 4 | 5 | 6 | 7 | 8 | 9 |
| Please provide more explanation for your rating |  | | | | | | | | |
| Are you more concerned about adverse effects from PPE use or EVD transmission? | 1. I am more concerned about adverse effects from PPE use than EVD transmission 2. I am more concerned about EVD transmission than adverse effects from PPE use 3. I am equally concerned about EVD transmission and adverse effects from PPE use | | | | | | | | |
| Why? |  | | | | | | | | |

Please suggest any additional outcomes that you perceive as important or critical for making the recommendation: _______________________________________________________________

______________________________________________________________________________

______________________________________________________________________________

### Part 2: Contextual factors

The development of recommendations is based on the consideration of both the effects of interventions on the outcomes of interest (i.e., efficacy and safety), and on the contextual factors. While the effects of interventions on the health outcomes of interest typically do not vary by context, the contextual factors do. Therefore, it is important to solicit the perspective of key interest-holders regarding contextual factors that may influence implementation including resource use, equity concerns, acceptability and feasibility.

In this exercise, we will ask you a series of questions about: A- PPE use; B- Decontamination and disinfection interventions. We are particularly interested to know whether these interventions are acceptable, whether these interventions are feasible, the resource use associated with those interventions, and whether there is an impact on equity associated with those interventions.

First, we will provide explanation for each of the contextual factors before we ask you to evaluate them in relation to the above interventions of interest.

Herein explanations of each of those contextual factors:

1. Acceptability

*Explanation:*

Acceptability of an intervention refers to the perception of whether it is appropriate, agreeable, tolerable, or satisfactory. Acceptability may be affected by the characteristics of the intervention as well as the person’s culture, preferences, beliefs and experiences related to the intervention.

1. Feasibility

*Explanation:*

Feasibility of an intervention refers to the extent to which it is successfully implemented within a given setting. Feasibility may be affected by the barriers and facilitators to implementing the interventions, resources needed (e.g., human resources), sustainability, availability, accessibility, and the potential for integration of an intervention within an existing health program.

1. Resource use

*Explanation:*

Resources associated with an intervention may be of three types: (1) health care resources (e.g., costs of the intervention, human resources, time, hospital visits and home visits); (2) non-heath care resources (e.g., social welfare services); and/or (3) patient and informal caregiver resources (e.g., time of family).

1. Impact on equity

*Explanation:*

Equity means all patients/people have equal and fair chance to receive the required intervention irrespective of their characteristics. Subgroups for whom equity might be particularly relevant are typically defined in relation to their place of residence, race/ethnicity/culture/language, occupation, gender/sex, religion, education, socioeconomic status, and social capital.

When assessing equity, consider how the implementation of the recommendation impacts the health equity of any of the above defined subgroups compared with the current status. Consider specifically whether it could improve health equity or cause or exacerbate existing inequities. Potential influencing factors include affordability and accessibility to the intervention to specific subgroups, and whether the benefits might be less for specific subgroups.

**I. Use of Personal Protective Equipment**

From your point of view, please appraise resource use, impact on equity, acceptability, and feasibility of the following PPE uses to reduce the transmission of Ebola in ETCs, other health care facilities and in the communities:

| 1.A These questions relate to the use of PPE whilst caring for a patient with EVD. In particular, whether health workers need to cover the head and neck (with a hood or head cover) AND mucous membranes (with a face shield/goggles and a mask) OR just cover the mucous membranes |
| --- |
| 1. Acceptability |
| Is covering the head and neck and mucous membranes compared to just covering the mucous membranes:   1. Less acceptable 2. Varies 3. More acceptable 4. Negligeable 5. Don’t know |
| Please explain your choice |
| Feasibility |
| Is covering the head and neck and mucous membranes compared to just covering the mucous membranes:   1. Less feasible 2. Varies 3. More feasible 4. Negligeable 5. Don’t know |
| Please explain why? And elaborate more on the facilitators and barriers for feasibility |
| 1. Resources (financial, human, and logistical) required for covering head and neck and mucous membrane |
| How does covering the head and neck and mucous membranes compared to just covering the mucous membranes compare in terms of cost:   1. More costly 2. No difference 3. Less costly 4. Negligeable 5. Don’t know |
| What are the resources needed for implementation? |
| Equity |
| Would there be groups of individuals (e.g., based on gender, religion, socioeconomic status, health care type, etc..) that may be unable to cover their head and neck and mucous membranes as compared to just covering the mucous membranes.   1. Yes 2. No 3. Don't know |
| If yes, please elaborate on who are those groups of individuals and why? |

| 1.B These questions relate to the use of PPE whilst caring for a patient with EVD. In particular, whether eye protection (goggles, face shield) should be worn under versus over the head and neck covering |
| --- |
| 1. Acceptability |
| Is using eye protection (goggles, face shield) under the head and neck covering compared to using eye protection over the head and neck covering:   1. Less acceptable 2. Varies 3. More acceptable 4. Negligeable 5. Don’t know |
| Please explain your choice |
| Feasibility |
| Is using eye protection (goggles, face shield) under the head and neck covering compared to using eye protection over the head and neck covering:   1. Less feasible 2. Varies 3. More feasible 4. Negligeable 5. Don’t know |
| Please explain why? And elaborate more on the facilitators and barriers for feasibility |
| 1. Resources (financial, human, and logistical) required for using eye protection (goggles, face shield) under vs over the head and neck covering |
| How does using eye protection (goggles, face shield) under the head and neck covering compared to using eye protection over the head and neck covering compare in terms of cost:   1. More costly 2. No difference 3. Less costly 4. Varies 5. Don’t know |
| What are the resources needed for implementation? |
| Equity |
| Would there be groups of individuals (e.g., based on gender, religion, socioeconomic status, health care type, etc..) that may be unable to use eye protection (goggles, face shield) under the head and neck covering compared to using eye protection over the head and neck covering?   1. Yes 2. No 3. Don't know |
| If yes, please elaborate on who are those groups of individuals, and why? |

I.C. Using disposable/biodegradable versus re-usable while providing direct or indirect care to Ebola patients.

| 1. Acceptability |
| --- |
| Using disposable/biodegradable versus re-usable while providing direct or indirect care to Ebola patients:   1. Less acceptable 2. Varies 3. More acceptable 4. Negligeable 5. Don’t know |
| Please explain your choice |
| Feasibility |
| Using disposable/biodegradable versus re-usable while providing direct or indirect care to Ebola patients:   1. Less feasible 2. Varies 3. More feasible 4. Negligeable 5. Don’t know |
| Please explain why? And elaborate more on the facilitators and barriers for feasibility |
| 1. Resources (financial, human, and logistical) required for using eye protection (goggles, face shield) under vs over the head and neck covering |
| Using disposable/biodegradable versus re-usable while providing direct or indirect care to Ebola patients:   1. More costly 2. No difference 3. Less costly 4. Varies 5. Don’t know |
| What are the resources needed for implementation? |
| Equity |
| Would there be groups of individuals (e.g., based on gender, religion, socioeconomic status, health care type, etc..) that may be unable using disposable/biodegradable versus re-usable while providing direct or indirect care to Ebola patients?   1. Yes 2. No 3. Don't know |
| If yes, please elaborate on who are those groups of individuals, and why? |

**II. Decontamination and disinfection**

| II.A. These questions relate to the disinfection of surfaces potentially contaminated with EVD virus, in particular whether spraying versus wiping surfaces with disinfectants. |
| --- |
| 1. Acceptability |
| Is spraying surfaces with disinfectant compared to wiping surfaces with disinfectant:   1. Less acceptable 2. Varies 3. More acceptable 4. Negligeable 5. Don’t know |
| Please explain your choice |
| Feasibility |
| Is spraying surfaces with disinfectant compared to wiping surfaces with disinfectant:   1. Less feasible 2. Varies 3. More feasible 4. Negligeable 5. Don’t know |
| Please explain why? And elaborate more on the facilitators and barriers for feasibility |
| 1. Resources (financial, human, and logistical) required for spraying vs wiping with disinfectant |
| How does spraying with disinfectant compared to wiping with disinfectant compare in terms of cost:   1. More costly 2. No difference 3. Less costly 4. Varies 5. Don’t know |
| What are the resources needed for implementation? |
| Equity |
| Would there be groups of individuals (e.g., based on gender, religion, socioeconomic status, health care type, etc..) that may be unable to spray or swipe with disinfectant?   1. Yes 2. No 3. Don't know |
| If yes, please elaborate on who are those groups of individuals, and why? |

| II.B. These questions relate to decontamination during the doffing of PPE, in particular spraying versus non-spraying of health workers. |
| --- |
| 1. Acceptability |
| Is spraying during doffing of PPE compared to non-spraying during doffing of PPE:   1. Less acceptable 2. Varies 3. More acceptable 4. Negligeable 5. Don’t know |
| Please explain your choice |
| Feasibility |
| Is spraying during doffing of PPE compared to non-spraying during doffing of PPE:   1. Less feasible 2. Varies 3. More feasible 4. Negligeable 5. Don’t know |
| Please explain why? And elaborate more on the facilitators and barriers for feasibility |
| 1. Resources (financial, human, and logistical) required for spraying during doffing vs non-spraying |
| How does spraying during doffing compared to non-spraying during doffing compare in terms of cost:   1. More costly 2. No difference 3. Less costly 4. Varies 5. Don’t know |
| What would be the resources needed for implementation? |
| Equity |
| Would there be groups of individuals (e.g., based on gender, religion, socioeconomic status, health care type, etc..) that may be unable to spray during doffing?   1. Yes 2. No 3. Don't know |
| If yes, please elaborate on who are those groups of individuals, and why? |

| II.C. These questions relate to performing hand hygiene in-between patients with EVD inside Ebola isolation/treatment centers |
| --- |
| 1. Acceptability |
| When caring for an Ebola patient which of the following options would be most acceptable for performing hand hygiene in between patients.   1. Wash/disinfect the outer glove between patients 2. Wash/disinfect the outer glove, remove it, and put on a new glove in-between patients. 3. Wash/disinfect outer glove, remove it, wash/disinfect inner glove, and put on a new outer glove in-between patients 4. Remove outer and inner glove, wash/disinfect hands, and put on new gloves |
| Please explain your choice |
| Feasibility |
| When caring for an Ebola patient which of the following options would be most feasible for performing hand hygiene in between patients.   1. Wash/disinfect the outer glove between patients 2. Wash/disinfect the outer glove, remove it, and put on a new glove in-between patients. 3. Wash/disinfect outer glove, remove it, wash/disinfect inner glove, and put on a new outer glove in-between patients 4. Remove outer and inner glove, wash/disinfect hands, and put on new gloves |
| Please explain your choice |
| 1. Resources (financial, human, and logistical) required for performing hand hygiene in between patients |
| When caring for an Ebola patient which of the following options would be the costliest for performing hand hygiene in between patients.   1. Wash/disinfect the outer glove between patients 2. Wash/disinfect the outer glove, remove it, and put on a new glove in-between patients. 3. Wash/disinfect outer glove, remove it, wash/disinfect inner glove, and put on a new outer glove in-between patients 4. Remove outer and inner glove, wash/disinfect hands, and put on new gloves |
| Please explain your choice |
| Equity |
| Would you be concerned that some groups of individuals (e.g., based on gender, religion, socioeconomic status, health care type, etc..) may not be able to use any of the above options for performing hand hygiene in between patients?   1. Yes 2. No 3. Don't know |
| If yes, please elaborate on who are those groups of individuals, and why? |

| II.D. These questions relate to the product that should be used to perform hand hygiene in-between patients with EVD inside Ebola isolation/treatment centers Washing and disinfecting using soap & water, alcohol-based hand rub (ABHR) or chlorine |
| --- |
| 1. Acceptability |
| When caring for an Ebola patient which of the following options would be most acceptable for washing and disinfecting in-between patients.   1. Wash with soap and water 2. Disinfect with Alcohol Based Hand rub 3. Disinfect with chlorine |
| Please explain your choice |
| Feasibility |
| When caring for an Ebola patient which of the following options would be most feasible for washing and disinfecting in between patients.   1. Wash with soap and water 2. Disinfect with Alcohol Based Hand rub 3. Disinfect with chlorine |
| Please explain your choice |
| 1. Resources (financial, human, and logistical) required for washing and disinfecting in between patients |
| When caring for an Ebola patient which of the following options would be the costliest for washing and disinfecting in between patients.   1. Wash with soap and water 2. Disinfect with Alcohol Based Hand rub 3. Disinfect with chlorine |
| Please explain your choice |
| Equity |
| Would you be concerned that some groups of individuals (e.g., based on gender, religion, socioeconomic status, health care type, etc..) may not be able to use any of the above options for washing and disinfecting in between patients?   1. Yes 2. No 3. Don't know |
| If yes, please elaborate on who are those groups of individuals, and why? |

| II.E. These questions relate to incinerating heavily soiled linen from patients versus disinfecting them |
| --- |
| 1. Acceptability |
| Is incinerating heavily soiled linen from patients versus disinfecting them:   1. Less acceptable 2. Varies 3. More acceptable 4. Negligeable 5. Don’t know |
| Please explain your choice |
| Feasibility |
| Is incinerating heavily soiled linen from patients versus disinfecting them:   1. Less feasible 2. Varies 3. More feasible 4. Negligeable 5. Don’t know |
| Please explain why? And elaborate more on the facilitators and barriers for feasibility |
| 1. Resources (financial, human, and logistical) required for incinerating soiled lined versus disinfecting them |
| Is incinerating heavily soiled linen from patients versus disinfecting them in terms of cost:   1. More costly 2. No difference 3. Less costly 4. Varies 5. Don’t know |
| What would be the resources needed for implementation? |
| Equity |
| Would there be groups of individuals (e.g., based on gender, religion, socioeconomic status, health care type, etc..) that may be incinerating versus disinfecting them?   1. Yes 2. No 3. Don't know |
| If yes, please elaborate on who are those groups of individuals, and why? |

| **Interview date** |  | | | | | | |
| --- | --- | --- | --- | --- | --- | --- | --- |
| **ID** |  | | | | | | |
| **Please indicate whether you are a:** | | | | | | | |
|  Doctor |  Nurse | |  Midwife | | |  Cleaner (hygienist) | |
| Community health worker | Laboratory personnel | | Other, please specify | | | | |
| **Please specify the type of organization you work in:** | | | | | | | |
|  Government   Non-governmental organization   UN | | | | | | | |
| **Experience in providing direct care to Ebola patients** | | | | | | | |
|  No |  Yes | | | | | | |
|  |  Please specify location:  ETC  Health care facility | | | | | | |
|  | **Please indicate your role in providing direct care to Ebola patients (check all that apply)** | | | | | | |
|  |  Collecting blood |  Screening and triage | |  Cleaning | | |  Transport |
|  |  Physical examination |  Clinical care | | |  Other, please specify | | |

## **Appendix 5:** Interview guide

**Introduction**

The World Health Organization (WHO) is currently in the process of developing guidelines on Infection Prevention and Control (IPC) measures to reduce the risk of transmission of EVD to health workers. Your perspective on these recommendations is important and will inform the guideline development group. We will ask a series of questions related to the importance of the outcomes of measures addressed in these guidelines and your perspective on factors that may influence the acceptability, feasibility, resource use, and if there are equity concerns.

| **Interview date** |  | | | | | | |
| --- | --- | --- | --- | --- | --- | --- | --- |
| **ID** |  | | | | | | |
| **Please indicate whether you are a:** | | | | | | | |
|  Doctor |  Nurse | |  Midwife | | |  Cleaner (hygienist) | |
| Community health worker | Laboratory personnel | | Other, please specify | | | | |
| **Please specify the type of organization you work in:** | | | | | | | |
|  Government   Non-governmental organization   UN | | | | | | | |
| **Experience in providing direct care to Ebola patients** | | | | | | | |
|  No |  Yes | | | | | | |
|  |  Please specify location:  ETC  Health care facility | | | | | | |
|  | **Please indicate your role in providing direct care to Ebola patients (check all that apply)** | | | | | | |
|  |  Collecting blood |  Screening and triage | |  Cleaning | | |  Transport |
|  |  Physical examination |  Clinical care | | |  Other, please specify | | |

| **Part 1 Valuation of outcomes** |
| --- |
| The main aim of infection prevention and control (IPC) measures is to prevent EVD transmission. From your perspective, how important is the **prevention of EVD transmission**?  Not important  Important  Critical  Don’t know |
| Please explain your choice |
| 1. Health workers may be exposed to chlorine whilst practicing IPC measures, how important are the **adverse effects of chlorine exposure**?   Not important  Important  Critical  Don’t know |
| Please explain your choice |
| **3**. Additional layers of PPE worn by health workers may lead to several short-term negative effects, how important are the **adverse effects from PPE use** to you?  Not important  Important  Critical  Don’t know |
| Please explain your choice |
| **4.** From your point of view, how concerned are you **about adverse effects from chlorine exposure** as compared to **transmission of Ebola**?   1. More concerned about transmission of Ebola than about adverse effects from chlorine exposure 2. Equally concerned about transmission of Ebola and adverse effects from chlorine exposure 3. Less concerned about transmission of Ebola than about adverse effects from chlorine exposure 4. Don’t know |
| Please explain your choice |
| **5.** From your point of view, how concerned are you about **adverse effects associated with PPE use** as compared to **transmission of Ebola**?   1. More concerned about transmission of Ebola than about adverse effects associated with PPE use 2. Equally concerned about transmission of Ebola and adverse effects associated with PPE use 3. Less concerned about transmission of Ebola than about adverse effects associated with PPE use 4. Don’t know |
| Please explain your choice |

**Part 2 Contextual Factors**

**I. Use of Personal Protective Equipment**

| I.A These questions relate to the use of PPE whilst caring for a patient with EVD. In particular, whether health workers need to cover the head and neck (with a hood or head cover) AND mucous membranes (with a face shield/goggles and a mask) OR just cover the mucous membranes |
| --- |
| 1. Acceptability |
| Is covering the head and neck and mucous membranes compared to just covering the mucous membranes:   1. Less acceptable 2. Varies 3. More acceptable 4. Negligeable 5. Don’t know |
| Please explain your choice |
| Feasibility |
| Is covering the head and neck and mucous membranes compared to just covering the mucous membranes:   1. Less feasible 2. Varies 3. More feasible 4. Negligeable 5. Don’t know |
| Please explain why? And elaborate more on the facilitators and barriers for feasibility |
| 1. Resources (financial, human, and logistical) required for covering head and neck and mucous membrane |
| How does covering the head and neck and mucous membranes compared to just covering the mucous membranes compare in terms of cost:   1. More costly 2. No difference 3. Less costly 4. Varies 5. Don’t know |
| What are the resources needed for implementation? |
| Equity |
| Would there be groups of individuals (e.g., based on gender, religion, socioeconomic status, health care type, etc..) that may be unable to cover their head and neck and mucous membranes as compared to just covering the mucous membranes.   1. Yes 2. No 3. Don't know |
| If yes, please elaborate on who are those groups of individuals and why? |

| I.B. These questions relate to the use of PPE whilst caring for a patient with EVD. In particular, whether eye protection (goggles, face shield) should be worn under versus over the head and neck covering |
| --- |
| 1. Acceptability |
| Is using eye protection (goggles, face shield) under the head and neck covering compared to using eye protection over the head and neck covering:   1. Less acceptable 2. Varies 3. More acceptable 4. Negligeable 5. Don’t know |
| Please explain your choice |
| Feasibility |
| Is using eye protection (goggles, face shield) under the head and neck covering compared to using eye protection over the head and neck covering:   1. Less feasible 2. Varies 3. More feasible 4. Negligeable 5. Don’t know |
| Please explain why? And elaborate more on the facilitators and barriers for feasibility |
| 1. Resources (financial, human, and logistical) required for using eye protection (goggles, face shield) under vs over the head and neck covering |
| How does using eye protection (goggles, face shield) under the head and neck covering compared to using eye protection over the head and neck covering compare in terms of cost:   1. More costly 2. No difference 3. Less costly 4. Varies 5. Don’t know |
| What are the resources needed for implementation? |
| Equity |
| Would there be groups of individuals (e.g., based on gender, religion, socioeconomic status, health care type, etc..) that may be unable to use eye protection (goggles, face shield) under the head and neck covering compared to using eye protection over the head and neck covering?   1. Yes 2. No 3. Don't know |
| If yes, please elaborate on who are those groups of individuals, and why? |

| I.C. Using disposable/biodegradable versus re-usable while providing direct or indirect care to Ebola patients. |
| --- |
| 1. Acceptability |
| Using disposable/biodegradable versus re-usable while providing direct or indirect care to Ebola patients:   1. Less acceptable 2. Varies 3. More acceptable 4. Negligeable 5. Don’t know |
| Please explain your choice |
| Feasibility |
| Using disposable/biodegradable versus re-usable while providing direct or indirect care to Ebola patients:   1. Less feasible 2. Varies 3. More feasible 4. Negligeable 5. Don’t know |
| Please explain why? And elaborate more on the facilitators and barriers for feasibility |
| 1. Resources (financial, human, and logistical) required for using eye protection (goggles, face shield) under vs over the head and neck covering |
| Using disposable/biodegradable versus re-usable while providing direct or indirect care to Ebola patients:   1. More costly 2. No difference 3. Less costly 4. Varies 5. Don’t know |
| What are the resources needed for implementation? |
| Equity |
| Would there be groups of individuals (e.g., based on gender, religion, socioeconomic status, health care type, etc..) that may be unable using disposable/biodegradable versus re-usable while providing direct or indirect care to Ebola patients?   1. Yes 2. No 3. Don't know |
| If yes, please elaborate on who are those groups of individuals, and why? |

**II. Decontamination and disinfection:**

| II.A. These questions relate to the disinfection of surfaces potentially contaminated with EVD virus, in particular whether surfaces should be sprayed versus wiped. |
| --- |
| 1. Acceptability |
| Is spraying surfaces with disinfectant compared to wiping surfaces with disinfectant:   1. Less acceptable 2. Varies 3. More acceptable 4. Negligeable 5. Don’t know |
| Please explain your choice |
| Feasibility |
| Is spraying surfaces with disinfectant compared to wiping surfaces with disinfectant:   1. Less feasible 2. Varies 3. More feasible 4. Negligeable 5. Don’t know |
| Please explain why? And elaborate more on the facilitators and barriers for feasibility |
| 1. Resources (financial, human, and logistical) required for spraying vs wiping with disinfectant |
| How does spraying with disinfectant compared to wiping with disinfectant compare in terms of cost:   1. More costly 2. No difference 3. Less costly 4. Varies 5. Don’t know |
| What are the resources needed for implementation? |
| Equity |
| Would there be groups of individuals (e.g., based on gender, religion, socioeconomic status, health care type, etc..) that may be unable to spray or swipe with disinfectant?   1. Yes 2. No 3. Don't know |
| If yes, please elaborate on who are those groups of individuals, and why? |

| II.B. These questions relate to decontamination during the doffing of PPE, in particular spraying versus non-spraying of health workers. |
| --- |
| 1. Acceptability |
| Is spraying during doffing of PPE compared to non-spraying during doffing of PPE:   1. Less acceptable 2. Varies 3. More acceptable 4. Negligeable 5. Don’t know |
| Please explain your choice |
| Feasibility |
| Is spraying during doffing of PPE compared to non-spraying during doffing of PPE:   1. Less feasible 2. Varies 3. More feasible 4. Negligeable 5. Don’t know |
| Please explain why? And elaborate more on the facilitators and barriers for feasibility |
| 1. Resources (financial, human, and logistical) required for spraying during doffing vs non-spraying |
| How does spraying during doffing compared to non-spraying during doffing compare in terms of cost:   1. More costly 2. No difference 3. Less costly 4. Varies 5. Don’t know |
| What would be the resources needed for implementation? |
| Equity |
| Would there be groups of individuals (e.g., based on gender, religion, socioeconomic status, health care type, etc..) that may be unable to spray during doffing?   1. Yes 2. No 3. Don't know |
| If yes, please elaborate on who are those groups of individuals, and why? |

| II.C. These questions relate to performing hand hygiene in-between patients with EVD inside Ebola isolation/treatment centers |
| --- |
| 1. Acceptability |
| When caring for an Ebola patient which of the following options would be most acceptable for performing hand hygiene in between patients.   1. Wash/disinfect the outer glove between patients 2. Wash/disinfect the outer glove, remove it, and put on a new glove in-between patients. 3. Wash/disinfect outer glove, remove it, wash/disinfect inner glove, and put on a new outer glove in-between patients 4. Remove outer and inner glove, wash/disinfect hands, and put on new gloves |
| Please explain your choice |
| Feasibility |
| When caring for an Ebola patient which of the following options would be most feasible for performing hand hygiene in between patients.   1. Wash/disinfect the outer glove between patients 2. Wash/disinfect the outer glove, remove it, and put on a new glove in-between patients. 3. Wash/disinfect outer glove, remove it, wash/disinfect inner glove, and put on a new outer glove in-between patients 4. Remove outer and inner glove, wash/disinfect hands, and put on new gloves |
| Please explain your choice |
| 1. Resources (financial, human, and logistical) required for performing hand hygiene in between patients |
| When caring for an Ebola patient which of the following options would be the costliest for performing hand hygiene in between patients.   1. Wash/disinfect the outer glove between patients 2. Wash/disinfect the outer glove, remove it, and put on a new glove in-between patients. 3. Wash/disinfect outer glove, remove it, wash/disinfect inner glove, and put on a new outer glove in-between patients 4. Remove outer and inner glove, wash/disinfect hands, and put on new gloves |
| Please explain your choice |
| Equity |
| Would you be concerned that some groups of individuals (e.g., based on gender, religion, socioeconomic status, health care type, etc..) may not be able to use any of the above options for performing hand hygiene in between patients?   1. Yes 2. No 3. Don't know |
| If yes, please elaborate on who are those groups of individuals, and why? |

| II.D. These questions relate to the product that should be used to perform hand hygiene in-between patients with EVD inside Ebola isolation/treatment centers Washing and disinfecting using soap & water, alcohol-based hand rub (ABHR) or chlorine |
| --- |
| 1. Acceptability |
| When caring for an Ebola patient which of the following options would be most acceptable for washing and disinfecting in-between patients.   1. Wash with soap and water 2. Disinfect with Alcohol Based Hand rub 3. Disinfect with chlorine |
| Please explain your choice |
| Feasibility |
| When caring for an Ebola patient which of the following options would be most feasible for washing and disinfecting in between patients.   1. Wash with soap and water 2. Disinfect with Alcohol Based Hand rub 3. Disinfect with chlorine |
| Please explain your choice |
| 1. Resources (financial, human, and logistical) required for washing and disinfecting in between patients |
| When caring for an Ebola patient which of the following options would be the costliest for washing and disinfecting in between patients.   1. Wash with soap and water 2. Disinfect with Alcohol Based Hand rub 3. Disinfect with chlorine |
| Please explain your choice |
| Equity |
| Would you be concerned that some groups of individuals (e.g., based on gender, religion, socioeconomic status, health care type, etc..) may not be able to use any of the above options for washing and disinfecting in between patients?   1. Yes 2. No 3. Don't know |
| If yes, please elaborate on who are those groups of individuals, and why? |

| II.E. These questions relate to incinerating heavily soiled linen from patients versus disinfecting them |
| --- |
| 1. Acceptability |
| Is incinerating heavily soiled linen from patients versus disinfecting them:   1. Less acceptable 2. Varies 3. More acceptable 4. Negligeable 5. Don’t know |
| Please explain your choice |
| Feasibility |
| Is incinerating heavily soiled linen from patients versus disinfecting them:   1. Less feasible 2. Varies 3. More feasible 4. Negligeable 5. Don’t know |
| Please explain why? And elaborate more on the facilitators and barriers for feasibility |
| 1. Resources (financial, human, and logistical) required for incinerating soiled lined versus disinfecting them |
| Is incinerating heavily soiled linen from patients versus disinfecting them in terms of cost:   1. More costly 2. No difference 3. Less costly 4. Varies 5. Don’t know |
| What would be the resources needed for implementation? |
| Equity |
| Would there be groups of individuals (e.g., based on gender, religion, socioeconomic status, health care type, etc..) that may be incinerating versus disinfecting them?   1. Yes 2. No 3. Don't know |
| If yes, please elaborate on who are those groups of individuals, and why? |

## **Appendix 6:** Framework thematic analysis

The proposed analytical approach consisted of 7 stages (Gale, 2013) .

- In stage 1, the audio-recording was transcribed verbatim and was translated into English if discussion was conducted in French.
- In Stage 2, two members of the research (GHA and JK) team familiarized themselves with the content of the discussion by reading each transcript and taking notes.
- In stage 3, the data coding started. At this stage, the data was indexed based on the EtD framework and labeled for each meaningful data.
- In stage 4, after indexing a few transcripts, the two members of the research team met to discuss their labeling, merged labels, and created categories thus creating an analytical framework for coding the remaining transcripts.
- In stage 5, they applied the analytical framework to the remaining transcripts.
- In stage 6, they charted the data into the analytical framework. Charting meant to allocate a category for each data statement from each participant. The chart included the perspectives of participants as illustration quotations indexed under a category.
- In stage 7, the researchers compared and contrasted the categories and mapped connections between them and shared them with the rest of the team for discussion. The resulting findings were meant to interpret the findings in view of the EtD framework.

Two members of the research team (GHA & JK) conducted the thematic analysis.

## **Appendix 7:** Survey participants’ characteristics (N=73)

|  | **Survey participants,**  **n (%)*** |
| --- | --- |
| **Female** | 32 (44) |
| **Healthcare worker group** |  |
| Physician | 35 (48) |
| Nurse | 19 (26) |
| Hygienist | 6 (8) |
| Laboratory worker | 3 (4) |
| Other** | 10 (14) |
| **Type of organization** |  |
| Health-care facility | 29 (40) |
| Governmental organization | 22 (30) |
| Non-governmental organization | 22 (30) |
| **Experience in providing direct care to patients with E**BOD | 43 (59) |
| **Setting of providing direct care to patients with EBOD** (n= 43) |  |
| Ebola treatment center/unit | 24 (56) |
| Health-care facility | 15 (35) |
| Other | 4 (9) |

*Numbers do not always add to 73 due to missing data. Percentages represent valid percentages.

** i.e., public health professional, epidemiologist, microbiologist, pharmacist, dentist, Water, Sanitation and Hygiene (WASH) specialist, infection prevention and control (IPC) coordinator.

## **Appendix 8:** Outcomes valuation

| **Outcome** | **Survey result** | **Exemplary quote** |
| --- | --- | --- |
| **Ebola virus transmission** | Critical:90%  **Fatal and transmissible**        **Impact on the health care system**      **Impact on the HCW’s family** | *“fatal disease”* _(P07)_; *“infect other patients”* _(P08)_; *“infect the neighbor and the outbreak will spread into the community”* _(P05)._  *“because if health-care workers are not going to work... that's another big problem” _(P13)._*    *“If one member of the family is affected, the rest of the family is affected.. For 21 days, they cannot go out to work, which affects the income and the well-being of the family” _(P16)._* |
| **PPEs’ adverse effects** | Critical (38%) | *“I want to say critical. Because it, I have been before working in the tent where you are sweating your goggles have steamed up. You can't see what you're doing, and you're dealing with very sick people, so you need to urgently do things and you can't see and I have wanted before to just take my goggles off and just be like, I've had enough.. It's not only discomfort, it's major discomfort and it's horrible” _(P11)._* |
| **Chlorine adverse events** | Critical (49%) & 33% important.  **Repetitive use**              **The concentration and product used** | *“One has to be cognizant that it is tough on people _(P02)._*  *“ The repetitive use of chlorine, can lead to airways disease or asthma. Whether they have a baseline sort of dermatitis to contend with or you know if through repetitive use day after day after day after day, you do induce bit of a dermatitis”* *(P10).*  *“Also the major concern is that often times the chlorine concentration used in ETC is not 0.05% but rather 0.5% and sometimes it is powdered chlorine, a highly corrosive substance”* *_(P13)._* |
| Comparison between EBOD transmission, adverse events of PPEs and chlorine | EBOD transmission is more important than adverse effects of PPE (53%) and adverse effects of chlorine exposure (58%).  **EBOD is fatal, adverse events are reversible**    **Benefits of PPE and chlorine outweigh their harms**            **EBOD transmission is equally important than adverse effects of PPE chlorine exposure** | *‘The horribleness of having Ebola and what people can go through... also, transmission is dangerous; while I can’t transfer chlorine exposure’ _(P03)_*        *“I will bear the side effects of the chlorine exposure in order to protect myself because you know, for Ebola there is no there is no, there is no actual medication.   Once you get the virus, it's all about your body to fight. I'd rather have this short-lived effect then ending my life”* *_(P08)_*  ‘*It is major discomfort... It is horrible... It is occluding my sight. Even dealing with little things... You want to touch your face... I find it extremely uncomfortable to deal with those adverse events... At times, I am at more risk because I am messing up with my PPEs. I have done things that shouldn’t have been done’* *_(P03)_* |

## **Appendix 9:** Contextual factors influencing PPE use

| **PPE measure** |  |  | Survey | Exemplary quotes |
| --- | --- | --- | --- | --- |
| I.A. Covering head and neck (with a hood or head cover) and mucous membranes versus only covering mucous membranes. | Resource use | More cost | 49% | *“Cost of PPEs..., disposing off the PPEs add more waste to manage” _(P05)._* |
|  |  | Negligible difference | 17% | " *I don't think there is a change in costs… many of the PPEs that we receive have hoods and their cover* " *_(P13)_* |
|  |  | Less cost | 20% | --- |
|  | Equity concerns | No | 51% | --- |
|  |  | Yes | 49% | ***Economy*** *“Certain countries may not be able to access the same quality of PPE if the Ministry of Health is handling procurement, leading to inconsistent supply and potential challenges in protection levels” _(P02)._*  ***Religious head covers****“Religious head coverings add an extra layer, which can be uncomfortable due to heat, but most women adapt by removing their head covering in designated areas before putting on PPE” _(P05)_*  ***Women’s HCWs hair style*** *“Some people may have very thick hair, making it difficult to fit into the coverall and hood” _(P05)._*  ***Rurality*** *“Availability in remote areas can be inconsistent” _(P13)._* |
|  | Acceptability | More acceptable | 71% | ***Enhanced feeling of safety*** *“The head-and-neck covering ensures complete protection and enhances the feeling of safety” _(P01)._* |
|  |  | Less acceptable | 29% | ***Patients’ fear*** *“They [patients] sometimes are afraid to see healthcare workers just in the full gear. People coming to them and even the parent family of the patient get afraid” _(P05)._*  ***Influences the delivery of care****: “it impairs tolerability and comfort to the healthcare worker and it eventually influences the amount of care that can be provided to patients” _(P10)._*  ***Does not address the mechanism of transmission:*** *“I think it's less acceptable because it transmission mechanism doesn't occur in intact skin _(P10)._* |
|  | Feasibility | More feasible | 61% | ***Easy to done***  *“Covering head and neck is not complicated, can be done easily and it gives a sense of safety*  *‘Even if takes more time, it protects better’ _(P02)_* |
|  |  | Less feasible | 19% | ***PPE*** *“Hoods that come with coveralls often don’t cover the whole face” _(P01)_*  ***Discomfort:*** *“More layers lead to sweating and discomfort, impacting patient communication” _(P07)_*_._  ***Time consuming between patients:*** *“Wearing a mask under the hood is time-consuming” _(P01)_*_._ |
| I.B. Wearing eye protection (goggles or face shield) under versus over the head-and-neck covering | Resource use | Varies | 11% | ---- |
|  |  | Negligible difference | 50% | *“You use the same materials and for us, the hood is the oldest disposable, and the goggles are reused and disinfected, so there’s no cost difference whether they are used under or over the hood” _(P01)._* |
|  |  | Less cost | 11% | ---- |
|  |  | Don’t know | 12% | ---- |
|  | Equity concerns | No | 52% | *“There is no problem with equity when it comes to under as compared to over” (P04).* |
|  |  | Yes | 12% | ***Wearing glasses*** *“For those who wear glasses, they often have to put their glasses on first and then secure them with adhesive to prevent them from falling off when wearing the head cover”* *_(P01)._*  **Hearing aids** “*Hearing problem you never know cause normally some of I know of two of the hearing devices plugged into the ear*” *_(P08)._*  ***Hairstyle:*** *“Having it under might be a challenge, especially during doffing when you want to avoid hair getting caught” _(P12)._* |
|  | Acceptability | More acceptable | 49% | ***Safety*** *‘The dangerous part is the mucous membrane and it makes more sense to keep it last when doffing everything out’ _(P03)._* |
|  |  | Less acceptable | 24% | ***Wearing eye glasses*** *‘Because I have glasses I prefer it over.. I adjusted it with the band.. Initially it is not comfortable and then you get used it’ _(P04)_*. |
|  |  | Varies | 14% | ***Availability of resources***  *“We used to have anti-fog products that we were putting in the goggles and this will be preventing those goggles from getting foggy”* *_(P03)._* |
|  | Feasibility | More feasible | 45% | ***Standard practice and ease of use:*** *"It's because that's what I was doing" (P12).*  ***Overall protection:*** *“The only negative about doffing it last is you can’t see very well…this is a barrier. But compared to alternative you can accidentally get Ebola in your mucous membrane.. I would rather have it under… Under is definitely better’’ _(P03)_* |
|  |  | Less feasible | 35% | ***Face shield/goggles adjustment:*** *“More feasible to put it over because I can see very well.. Under is so foggy and cloudy” _(P04)_* |
|  |  | Varies | 9% | ***Training:*** *"As long as you've been trained properly, it's feasible either way" _(P02)._* |
| I.C. Use of disposable/biodegradable versus reusable aprons | Resource use | More cost | 48% | *“More costly, quite drastically more costly with the disposable... Logistically, you need to find a distributor, different grades, sometimes they are great aprons, other times it is the thinnest plastic and it is flimsy... So in that sense it is more expensive…” _(P03)._* |
|  |  | Negligible difference | 14% | *“Disposable are less expensive but every time you are doing a procedure, you use one and dispose it so you can use 6-7 per day….. At the economic level, I find the re-usable more feasible.. But you also need to disinfect the re-usable… Waste management is costly.. You need incinerators” _(P02)._* |
|  |  | Less cost | 17% | *“Well, we look at the cost of disinfection, treating treatment, we might say reusable is expensive” _(P04)._* |
|  | Equity concerns | No | 64% | *“There is no reason to be concerned about inequity” (P11).*  *“Aprons are accessible to everyone, it's not a problem” _(P16)._* |
|  |  | Yes | 16% | ***Low resource settings:*** *"Some health facilities might not have incinerators or good waste management systems, making disposable aprons problematic" _(P05)._*  ***Rurality:*** *"In remote areas, the availability of both disposable and reusable aprons is a significant equity issue due to the logistical challenges of cleaning and drying" _(P13)._*  ***The task assigned to the HCW:*** *“cleaners need to use disposable for example to clean the vomit” _(P04)._*  ***Size and fit***: *“For smaller persons, heavy reusable aprons are a bigger challenge because they are heavy and big, and don’t come in different sizes, which means they might drag along the floor” _(P12)._* |
|  | Acceptability | More acceptable | 68% | ***Guarantee cleanliness and eliminate the need for disinfection***. *“I think it’s probably more acceptable wearing the disposable ones... because like when something is disposable, yeah example you’re apron might be infected with the virus or contaminated with blood or whatever something of the patient, and when you throw it away, the health care worker just wears a new apron, so it’s guaranteed that the apron is disinfected, and that it’s clean, and when you use it, uhm” _(P06)._*  ***Weight and safety:*** *“I think it’s; it will be more acceptable because it's lighter… It even gives you more sense of safety because you know once you remove it, it's not coming back” _(P06)._*  ***Cleaning:*** *“It is not a good idea to use reusable... They need to be cleaned and back to the patient care... If not cleaned properly, then risk of transmission of virus” _(P03)._*  “*Reusable may not be totally safe. I don’t trust the cleaners… Maybe they are not disinfecting properly... They can make you feel hot” _(P04)._* |
|  |  | Less acceptable | 17% | ***Fragile reusable:*** *“Sometimes they can rip,, nothing is perfect” _(P04)_*  *“The disposable by the grey ball? Yeah, definitely they rip, so they're not great” _(P11)._* |
|  | Feasibility | More feasible | 67% | ***Comfort:*** *“More feasible, because they are more comfortable” _(P04)_*  ***Manageability:*** *“Because if you re-usable, you need to disinfect them. For the disposable you just put them in a special bin… More feasible then re-usable” _(P01)_*  ***Less exposure to chlorine:*** *“re-usable more exposure to chlorine” _(P01)_*    ***Easier to procure*** *“A pack of disposable ones will have about 100 aprons, which are discarded after use, and they are cheap” _(P09)._*  ***Availability:*** *“Disposable are more available’ (P02) ‘If you don’t have many re-usable ones and you need one another disinfected, so the supply is important” _(P05)_* |
|  |  | Less feasible | 18% | ***Waste management:*** *“When you throw it away”. _(P01)_*  ***Environmental concerns:*** *“I can think of good things in that it's good for the environment” _(P11)._* |

## **Appendix 10:** Contextual factors influencing decontamination and disinfection

|  |  |  | Survey | Exemplary quotes |
| --- | --- | --- | --- | --- |
| II.A. Spraying versus wiping surfaces with disinfectant | Resource use | More cost | 42% | **Larger amount of disinfectant:** *"The spraying of course is costly, I can see. Because from experience you could see the number of those sprayers, those were used and broken, you have to replace every time. So, it cost a lot the sprayers, it cost a lot"* _(P03)._  **Trained personnel:** *"Spraying is again more costly...it needs experts...we may need to have trained personnel...and all the logistics that are involved...will add additional cost "_(P07)._* |
|  |  | Negligible difference | 22% | *"The same there's no cost, there's no difference. Okay, the only thing. For the spraying, you need to have that spray...but you buy it at once. Then the wiping must have the wiping material. So, no difference" _(P08)_.* |
|  |  | Less cost | 14% | *“Wiping is more expensive. First, you need tissue, disposable, chlorine, Spraying can reach anywhere. Wiping takes time, they had to be thorough. More disinfectant...” _(P05)._* |
|  | Equity concerns | No | 59% | *“I don't think there are differences...”_(P12)._* |
|  |  | Yes | 17% | ***Short individuals:*** *“Short people... will not be used to using the canister” _(P04)._*  ***Safety and proximity*** *"Wiping with disinfectant is mainly looked at as it feels like it's not protective because for spraying you have a distance you can actually spray at a distance of 1 meter or something, and therefore somebody may feel safe spraying from a distance” _(P09)._*  ***Socioeconomic factors:*** *“It's expensive... you have to use a tissue which is disposable... there’s a problem with socio-economic status... access to these materials” _(P05)._*  ***Individuals at risk:*** *“You know you're spraying some with chemicals, so people with asthma and things like that, I can't imagine it. It would be very fair to spray them with chemicals, so people with health problems to do with their lungs and respiratory tracts and stuff, I would say not great to spray them or people with skin problems spraying them with chemicals is probably not a. Great thing to do. Not very fair” _(P11)._* |
|  | Acceptability | More acceptable | 25% | ***Surface coverage: “****I mean, uneven... some surfaces like... the back of a somewhat rusty ambulance... you're just going to have to coat the surface if you're actually trying to disinfect with something” _(P10)._* |
|  |  | Less acceptable | 67% | ***Belief wiping being more effective***. “*Wiping is better. Spraying maybe some areas not covered, missing spots.. (P06); ‘Wiping is more effective, will “physically” remove the virus”_(P01)._*  ***Splashing:*** *"Yeah, for me to wipe is OK because at least we avoid the splash " (P03);  ’Generates aerosols which have potential for inhalation of germs and disinfectant chemicals” _(P05_****_)_.***  ***Wiping:*** “*Wiping enables disinfectant to stay longer on surfaces...If something is contaminated with blood, you don’t spray, you wipe” _(P01)_* |
|  |  | Varies | 7% | *“If I was the cleaner I would rather spray.. Wiping is good for hard to reach area.. “ _(P03)_*. |
|  | Feasibility | More feasible | 49% | ***Easier to perform, less physical effort, and less time consuming*** *“It's easier to spray than to wipe... it takes more time and effort to wipe than it does to spray” _(P11)._ "When you look at the human resource and time... it's easier to spray than to wipe"* *_(P13)._* |
|  |  | Less feasible | 41% | *“Wipes are easily used and stored…*  *Wiping you use a cloth soaked in chlore.. Manual and doesn’t need reparation of equipment” _(P02)_* |
|  |  | Varies | 7% | *“After spraying you need to wipe”  _(P04)_* |
| II.B. Spraying versus non-spraying of health workers during doffing of PPE | Resource use | More cost | 56% | *“Yeah, I think it increases finances now. Yes, it increased financial, human and logistical results because more money is needed for the spray and whatever which is going to be used, the person has to be appointed to do the job, to do to spraying job, especially for us in the third, third world. And since we don't have a, we don't have a vacuum room. And then of course logistically, because you have to buy all those things. So, I think all resources are affected” _(P06)._*  **Increased risk:** “*More costly.. It also makes people sick, so you are loosing people at work” _(P03)_* |
|  |  | Negligible difference | 18% | *----* |
|  |  | Less cost | 9% | *“Spraying is less costly when compared to the benefits of avoiding the disease” _(P02)_* |
|  | Equity concerns | No | 49% | ---- |
|  |  | Yes | 21% | ***Individuals with health problems***: *“The allergy when you are spraying... you are dispersing the particles in the air even for environmental control measures... you will start coughing or sneezing” _(P05)._*  ***Spraying could increase transmission:*** *“It makes me uncomfortable when I’m getting dripped down over my eyes and my nose after coming out” _(P10)._*  ***Unequal protection:*** *“There are people who cannot be sprayed, for example, those working in treatment centers with disposable PPE who were not in direct contact with the sick” _(P16)._  “Cleaners for example they are not sprayed” _(P02)._* |
|  | Acceptability | More acceptable | 35% | ***Safety:*** *“I believe part of the virus is being eradicated their skills, some of it” _(P04)._ “Safety, to be sure that you haven't carried anything and in case you've carried by mistake, maybe in the gun modes everything because they remove, they remove and they put they spray and then they leave them in the red zone. So, then they have to move to the green zone without all that in contaminated stuff” _(P08)._* |
|  |  | Less acceptable | 57% | *“Spraying is less acceptable. You don’t need to do it... It is a horrible experience.  It is much nicer without spraying... The reason is to prevent contaminating with Ebola but if you are doffing correctly you don’t need to be sprayed, which is toxic and inhale... If you are doffing safely using correct technique, there is no reason to be sprayed... In anyway, you need to take a shower and wash everything before you get away. I don’t understand the need to spray.” _(P03)_. “Less acceptable to spray than non-spraying. From those who were sprayed in the field. Spraying does not reach anything. There is no additional benefit. There is no need for spraying... You had a good hygiene before you doff, no need for spraying” _(P06)_.*  ***Increase risk of transmission of EBOD:*** *“For example, I don’t think it was acceptable. You need to protect the mucous membrane while spraying _(P01)._ ‘When you spray, you take viruses from one place to another. You are likely to contaminate yourself” _(P05)_*. |
|  |  | Varies | 3% | *--*-- |
|  | Feasibility | More feasible | 38% | ---- |
|  |  | Less feasible | 56% | *“We don’t have the basin to collect the contaminated water, it is not feasible because we have disposable and it is time consuming” _(P01)._*    *“Not easier. There is someone is spraying you” _(P03)_ “Logistically, it's easier without the spraying because it's one less part of the package… and because you need more steps” _(P12)._* |
|  |  | Varies | 3% | *----* |
| II.C. Performing glove disinfection hand-hygiene between patients following either of four approaches | Resource use | More cost | 70% | *“This is the costliest... You use more gloves, and you are risking yourself, which is very expensive” _(P02)._* |
|  |  | Negligible difference | - | *----* |
|  |  | Less cost | - | ---- |
|  | Equity concerns | No | 63% | ---- |
|  |  | Yes | 19% | ***Size:*** *“the gloves are available except for the extreme sizes, those people who are huge, it also cuts across those people who have bigger sizes, there are issues” _( P11)_.*  *“Sometimes they run out of that size or they don't have small sizes, they just have the larger sizes and then this is a problem because look, you know if the glove is too small then then it's very uncomfortable and it can tear. And if the glove is too large, then it. Can fall off so you know you have to have gloves at the right size” _(P12)_*.  **Allergy:** *“Other people are allergic to the texture of gloves” _(P13)_.*  ***Discomfort and double gloving:*** “*People complain about double gloving because sometimes there's a lot of heat in the hands that comes with double gloving and also the doffing process of double gloving many times for many health workers, it's quite cumbersome, because while they are trying to remove the outer glove, the inner one also comes off” _(P09)._*  ***Accessibility:*** *“We've seen villages where latex gloves, eh? Of course. They wash the dirty ones to reuse them. Because they say it's been a year since they've received gloves xx... you have to import them so that they're accessible, but also educate them about the risks they run when they do these practices" _(P14)._*  ***Type of work:*** *“there are people who will be at a disadvantage. For example, a carpenter or cashier might refuse to wear gloves because they find them uncomfortable or too hot to wear all day”* *_(P16)._* |
|  | Acceptability | More acceptable |  | *“You wash and you go to another patient.. In the ETC, you cannot remove any protection” _(P02)._*  “ *so you remove the outer glove, if you remove properly the probability of infecting the inner glove is prevented”  _(P02)._*  *“Disinfecting ruins the glove, just remove them” _(P05)._*  *﻿“The inner gloves, it becomes wet and that it makes it very, very hard to wear a new glove on a wet glove” _(P06)._* |
|  |  | Less acceptable | -- | *----* |
|  | Feasibility | More feasible | -- | Option (a):  “*It is not cumbersome and not dangerous”_(P02)._*  *Option (b): “Because you can only remove the outer glove because the other is inside the gown, as long as the inner glove is intact” _(P06)._* |
|  |  | Less feasible | -- | *----* |
| II.D. Using soap and water versus ABHR versus chlorine for hand hygiene | Resource use | More cost | 58% | *"The alcohol-based hand rub would be a good option but it's expensive. It's very expensive to have alcohol-based hand rub full time in the ETC. I think we would go through several liters, and unless there's a lot of production happening close by"* *_(P09)._*  *"It's costlier and because it's made of alcohol, alcohol is a volatile solvent. It's easily dried up so they refill and refill and refill it every time" _(P04)._* |
|  |  | Less cost | 15% | *"Chlorine is probably the cheapest one just because of the way that you can produce it on such large volumes" _(P12)._*  (*"one quantity of hand sanitizer can buy you 3 to 4 quantities of soap"* *_(P13)._* |
|  | Equity concerns | No | 56% | *----* |
|  |  | Yes | 25% | ***Accessibility:*** *"In terms of equity, it is unbalanced, there is a large category that does not have access to chlorine. There are even places where chlorine only comes when there is an epidemic. If there is no epidemic, there is no source of supply. Hydroalcoholic gel is the same thing, it is expensive, it is much more for VIP structures, VIP health structures, health structures xx a little high xx framework, but the average population, the common people, they are more water and soap, it is much more accessible” _(P14)._*  ***Water scarcity:*** *"Some places the water is not enough or whatever, so they try to manage and keep chlorine for a longer period" _(P03)._*  ***Availability of ABHR:*** *"Alcohol-based hand rub is the most difficult to come by... there's probably an equity issue around having that available" _(P10)._*  ***Allergy:*** *“Those who are allergic to soap and those who are allergic to chlorine disinfectant” _(P16)._*  *Religious:* “*So even with some healthcare workers. So, their religion bound them to prohibit the use of anything that has alcohol content. So, based on that even when they are applying it on the skin, not consuming it, but they kick back against the use of alcohol. This and because of its alcoholic content”* *_(P07)._* |
|  | Acceptability | More acceptable | 45% (ABHR)  34% (Chlorine)  21% (water | ***ABHR:*** *“This hand sanitizer can eradicate all pathogens” (P04). ‘The virus is an envelope virus so the alcohol will kill the virus. And doesn’t have the side effects of chlorine. Soap and water you need to dry it,, ABHR does not need to dry and convenient. Soap and water needs to treatment” _(P06)._*  ***Odor (ABHR):*** *“The smell of alcohol-based hand rub is less pungent compared to chlorine”* *_(P09)._*  ***Gentle on skin (ABHR):*** *“Alcohol-based hand rub is good because it’s easier on the skin and gloves” (P06)* and *“if you use chlorine it is corrosive and it can damage the glove” _(P04)._*  ***Chlorine:*** “*health workers feel safer disinfecting with chlorine when they have gloved ones, and this has been based on memory and the kind of training that we received, especially from the outbreak in Liberia, Sierra Leon and Guinea. … there would be no need for soap and water if you have chlorine” _(P09)._* |
|  |  | Less acceptable | -- | *“Soap and water is messy and it will splash everywhere and it takes time, thus risky” _(P04)._* |
|  | Feasibility | More feasible | 38% (ABHR)  37% (Chlorine)  25% (water) | ***Quick to use (ABHR):*** *“The reason is easy, and you do not waste time” _(P05)._*  ***Availability and ease of use (Chlorine):*** *“Yes, because it's more feasible of course, it is more feasible. It's available anytime”* _(P03)._ |
|  |  | Less feasible | -- | ***Skin irritation (Chlorine):*** *“Yeah, you can use it but for me, from what I've seen in the use of chlorine, there are some patients who were developing a kind of rash or whatever, due to the use of chlorine” _(P03)._*  ***Concentration (Chlorine):*** *“...it's cumbersome to refill and it you see to make sure that there's always a 0.5% chlorine” _(P09)._* |
| II.E. Incinerating versus disinfecting heavily soiled linen from patients | **Resource use** | More cost | 56% | ***Equipment:*** *“You know to buy an incinerator. It's a huge money…The incinerator machine have different capacity. We have heavy duty. We have large space form. They don't have big sizes. But in case of Ebola I think they normally put the bigger incinerator very giant one” _(P04)._*  ***Electricity and technical expertise:*** *“in terms of hospitals... Us to do its high electricity consumption has to do with. Again, getting the overall maintenance and even the waste load again sometimes can override the capacity of the incinerator and people again who are more skilled or knowledgeable to be operating those machines as well can” _(P07)._*  ***Replenish linen:*** *“So, it it's costly again because at every given time you may for every given patient, you may need to replace them. Not even one patient, but for maybe, for instance, one patient might within a week you leaning soil leaning can be changed over how many times. So that replacement again is a problem. The supplies might not be readily available for changing of soiled linens so we can get”* *_(P07)._*  ***Waste management:*** *"Incineration goes with more cost, more costly to manage than disinfection"* *_(P07)._* |
|  |  | Negligible difference | 12% | *----* |
|  |  | Less cost | 19% | *----* |
|  | Equity concerns | No | 50% | *----* |
|  |  | Yes | 20% | ***Cleaning staff*** “*Yeah, I mean the problem with equity in this situation is when disinfecting is you're putting your cleaning staff. I think it not insignificant risk. And people who are doing your cleaning tend to be poor.” _(P02)._ “There re high risks of individuals who are handling those linens” _(P07)._*  ***Facilities and communities:*** “T*here are people who cannot incinerate laundry instead of disinfecting it, there are people who cannot do it…the law of their community prohibits that we can burn clothes for example of someone who loves in relation..we cannot burn the clothes of someone who died of Ebola” _(P16)._* |
|  | Acceptability | More acceptable | 59% | ***Effectiveness:*** *“When you incinerate the soiled linen, it's guaranteed that the virus is killed”* *_(P01)._*  ***Safety:*** *“Yeah, from the perspective of the healthcare worker, it is much more acceptable…you feel safer that the materials have been destroyed completely... Ebola has a huge stigma. So, you want to make sure you don’t want to affect the community”* _(P06)._  ***Exposure to disinfectants:*** *“So, this might be largely due to the leaks associated with the entire disinfection process "* _(P07)._ |
|  |  | Less acceptable | 35% | ***Wastage of linen: “****There is a cost to incinerating. If you have to incinerate every linen. Do you have the mean to replace the incinerated items”_(P05)_.*  *“In UK. Incinerating is less acceptable than disinfecting. It goes into the hospital linen system.. It is safe, this means we keep on using linen. It is safer to have it disinfected rather than burning and exposing the community” _(P03)_*  ***Risk to the community:***  *“Incinerating is not good for the community. If you are nearby, the person who does it who won’t have the best support and this is expected to be the job and they are at increased risk… The first and foremost to keep back the transmission” _(P03)._*  ***Logistical issues:*** *Because there's a lot of PPE that has to be incinerated at a go and it's costing a lot of fuel and sometimes you will find that some linen hasn't been incinerated that day because of the challenges” _(P09)._*  ***Safety****: “The dangers are in relation to all these toxic gases, CO2”* *_(P14)._*  ***Community:*** *“We have even a problem with this incinerating of patient clothes contaminated because the family of the patient have a close that he loved too much he doesn’t want to lose” _(P05)._* |
|  | Feasibility | More feasible | 58% | ***Accessibility:*** *“For incinerators, there is this locally made incinerators we use or they built. In all the big hospitals, referral hospitals we do have incinerators” _(P03)._*  ***Ease of use:*** *“I think it's more feasible. Uh, well, actually now that I think about. It easier to burn stuff probably than to scrub it” _(P02)._* |
|  |  | Less feasible | 29% | ***Collecting and transporting solid linen:*** *“For us incinerating is feasible however we have to collect the soiled linen in a special bin and transport it to a waste processor. So, before it gets incinerated it takes a while, there are a few steps in between” _(P01)._*  ***Rural areas:*** *“ETCs have like bonfire in the camp. Incinerator is rather a big word for the ones there. It is not great, someone has to burn everything, who knows the level of infection, the safety aspect is not good. In rural areas, you don’t have incinerators. NGO come and build generator and it broke and we don’t know how to fix it” _(P03)._*  ***Community:*** *“Incinerating is not good for the community. If you are nearby, the person who does it who won’t have the best support and this is expected to be the job and they are at increased risk…” _(P03)._* |
|  |  | Varies | 10% | *----* |
